# Point-of-care molecular testing and antiviral treatment of influenza in residents of homeless shelters in Seattle, WA: study protocol for a stepped-wedge cluster-randomized controlled trial

**DOI:** 10.1186/s13063-020-04871-5

**Published:** 2020-11-23

**Authors:** Kira L. Newman, Julia H. Rogers, Denise McCulloch, Naomi Wilcox, Janet A. Englund, Michael Boeckh, Timothy M. Uyeki, Michael L. Jackson, Lea Starita, James P. Hughes, Helen Y. Chu, on behalf of Seattle Flu Study Investigators

**Affiliations:** 1 Division of Allergy and Infectious Diseases, University of Washington, UW Medicine at South Lake Union, Chu Lab Room E630, 750 Republican St., Seattle, WA 98109 USA; 2 Seattle Children’s Research Institute, Seattle, WA USA; 3 Fred Hutchinson Cancer Research Center, Seattle, WA USA; 4 Influenza Division, Centers for Disease Control and Prevention, Atlanta, GA USA; 5 Kaiser Permanente Washington, Seattle, WA USA

## Abstract

**Introduction:**

Influenza is an important public health problem, but data on the impact of influenza among homeless shelter residents are limited. The primary aim of this study is to evaluate whether on-site testing and antiviral treatment of influenza in residents of homeless shelters reduces influenza spread in these settings.

**Methods and analysis:**

This study is a stepped-wedge cluster-randomized trial of on-site testing and antiviral treatment for influenza in nine homeless shelter sites within the Seattle metropolitan area. Participants with acute respiratory illness (ARI), defined as two or more respiratory symptoms or new or worsening cough with onset in the prior 7 days, are eligible to enroll. Approximately 3200 individuals are estimated to participate from October to May across two influenza seasons. All sites will start enrollment in the control arm at the beginning of each season, with routine surveillance for ARI. Sites will be randomized at different timepoints to enter the intervention arm, with implementation of a test-and-treat strategy for individuals with two or fewer days of symptoms. Eligible individuals will be tested on-site with a point-of-care influenza test. If the influenza test is positive and symptom onset is within 48 h, participants will be administered antiviral treatment with baloxavir or oseltamivir depending upon age and comorbidities. Participants will complete a questionnaire on demographics and symptom duration and severity. The primary endpoint is the incidence of influenza in the intervention period compared to the control period, after adjusting for time trends.

**Trial registration:**

ClinicalTrials.gov NCT04141917. Registered 28 October 2019. Trial sponsor: University of Washington.

**Supplementary information:**

The online version contains supplementary material available at 10.1186/s13063-020-04871-5.

## Introduction

Annual influenza epidemics are associated with high morbidity and mortality rates, especially among individuals who are elderly, chronically ill, or pregnant [1]. The Centers for Disease Control and Prevention (CDC) estimates that influenza has resulted in 140,000–810,000 hospitalizations and 12,000–61,000 deaths annually since 2010 [2].

### People experiencing homelessness as a high-risk group

As of 2018, approximately 12,000 people in Seattle are experiencing homelessness of whom 48% are housed in a shelter [3]. Homeless individuals experience higher morbidity and mortality than the general population, in part due to untreated or poorly controlled chronic medical conditions, infections caused by lack of access to sanitation, and high rates of mental illness and substance abuse [4]. Individuals in shelters may be at heightened risk for acquisition and transmission of influenza due to overcrowding, inadequate ventilation, and shared hygiene facilities [5–7]. In this population, chronic conditions are less likely to be controlled, increasing the risk of severe infections [8]. The CDC recommends initiation of antiviral therapy in high-risk outpatients with suspected influenza [9]. Despite a significant proportion of the homeless population qualifying as high risk from chronic comorbidities, studies have shown that these individuals encounter significant barriers to accessing testing and treatment services for acute infections [10]. Data concerning transmission of influenza within homeless shelters remain limited as well, with some cross-sectional studies showing high prevalence of respiratory illnesses in shelters.

### Acute respiratory illness (ARI) burden in people experiencing homelessness

Past studies have described local outbreaks of influenza and other respiratory viruses in homeless shelters [11]. A cross-sectional investigation of respiratory virus prevalence conducted in French shelters detected at least one pathogen in 8.7% of participants [7]. In a study of adults hospitalized in an urban hospital in Seattle during a 5-year period, people experiencing homelessness accounted for one third of individuals diagnosed with respiratory syncytial virus (RSV) but were only 10% of the overall hospitalized population [12]. A New York-based study of three shelter clinics found that people experiencing homelessness had high rates of pneumonia and pneumonia-related death [6]. Another study found pneumonia or influenza-related mortality rates among a cohort of adults experiencing homelessness aged 25 to 44 ranged from 11.9–36.6 per 100,000 person years, a rate ratio of 1.6–6.3 when compared to the general population [13].

### Testing and antiviral therapy accessibility

Early oseltamivir reduces duration of symptoms and lowers the risk of some complications among individuals with uncomplicated influenza [14–16]. Early oseltamivir treatment can also prevent secondary infections within households [17, 18].

Baloxavir marboxil is a newly approved oral agent for early treatment of uncomplicated influenza that functions as a cap-dependent endonuclease inhibitor, preventing influenza genome synthesis. It has similar clinical efficacy to oseltamivir but reduces viral load faster and is a single-dose regimen [19]. Therefore, baloxavir may have the potential for reducing person-to-person influenza virus transmission. Compliance with single dose baloxavir treatment of influenza is likely to be higher than a 5-day oseltamivir treatment course. Baloxavir is not approved for use in children under 12 years of age. However, oseltamivir is an approved option for the treatment of influenza in this age group.

### Rationale

There remain important unanswered questions regarding influenza burden and prevention of transmission in sheltered homeless populations. While prior studies have established that homeless populations are at high risk for tuberculosis, hepatitis A, and pneumonia, there are inadequate data regarding transmission of influenza and other respiratory viruses [20]. It is also unknown how a single-dose treatment with an antiviral such as baloxavir will impact incidence throughout a season in a densely populated community space like a shelter. Neither on-site point-of-care testing for respiratory pathogens nor on-site pharmaceutical treatment has been evaluated as a method of infection prevention in homeless shelters. Studies show that rapid molecular influenza tests are very sensitive and specific, yet there is an unmet need to evaluate their usefulness in a low-resource high-density community setting [21].

## Objectives

The objective of the trial is to evaluate efficacy of on-site point-of-care rapid influenza molecular testing and rapid antiviral treatment with baloxavir or oseltamivir in influenza-positive individuals for decreasing influenza incidence within homeless shelters.

### Primary outcome measure

The primary outcome will be the incidence of influenza in shelters during the intervention period compared to the incidence during the control period, after adjusting for underlying time trends. It will be calculated as the number of cases of laboratory-confirmed influenza among shelter residents per person-day of observation (person-days of observation will be based on the aggregate weekly census at the shelter). The incidence during intervention periods will be compared to the incidence during non-intervention periods using generalized linear mixed models to control for clustering by shelter and temporal variation.

### Secondary outcome measures

*Adherence and resource utilization outcomes*

○ Health resource utilization and school and work absenteeism among influenza cases

○ Total number of person-tests per census-day at shelters

○ Participant completion of administered study drug (only applicable to oseltamivir)

○ Loss-to-follow-up after on-site influenza diagnosis

*Clinical outcomes*

○ Symptom type, duration, and severity among influenza-positive cases

○ Clinical, demographic, and behavioral factors associated with asymptomatic influenza-positive cases

○ Relationship between symptom type, duration and severity, and seasonal influenza vaccination status

○ Asymptomatic fraction, i.e., probability of laboratory-confirmed influenza without meeting illness criteria

*Laboratory outcomes*

○ Semiquantitative viral load at day 0, day 2/3, and day 5/6/7

○ Proportion of follow-up samples from influenza-positive cases with detectable influenza virus by quantitative real-time polymerase chain reaction (qRT-PCR) at days 2/3 and days 5/6/7

○ Proportion of secondary influenza cases as identified via whole genome sequencing and sequence identity of 95%

○ Emergence of antiviral resistance, assessed by whole genome sequencing of influenza viruses and detection of PA/I38X and non-PA/138 substitutions for baloxavir [22, 23] and H275Y and other NA mutations for oseltamivir [24].

### Hypothesis

Our primary hypothesis is that implementation of an on-site point-of-care rapid molecular influenza diagnostic test and antiviral treatment intervention for influenza among sheltered individuals experiencing homelessness will reduce the incidence of influenza within this population.

## Methods/design

The protocol for this study is in accordance with Standard Protocol Items: Recommendations for Interventional Trials (SPIRIT) [25]. A SPIRIT checklist is provided in Additional file 1.

### Seattle Flu Study design

The Seattle Flu Study (SFS) is a multi-year surveillance study for influenza in the Seattle-metro area [26]. This current protocol is a nested sub-study within SFS.

## Study design

The trial is being conducted in nine homeless shelters in the Seattle, WA metropolitan area. Shelters were selected within Washington’s King County to include a diverse population in terms of age, sex, and race that was reasonably representative of Seattle’s homeless population and had large enough nightly capacities that we would likely achieve statistical power for our proposed intervention. The nine shelters house different populations including men, women, or families, and have maximum nightly populations between 45 and 212 individuals each, with a total maximum nightly population estimated at 1032 individuals. The University of Washington is the sponsor of the trial.

**Fig. 1:**
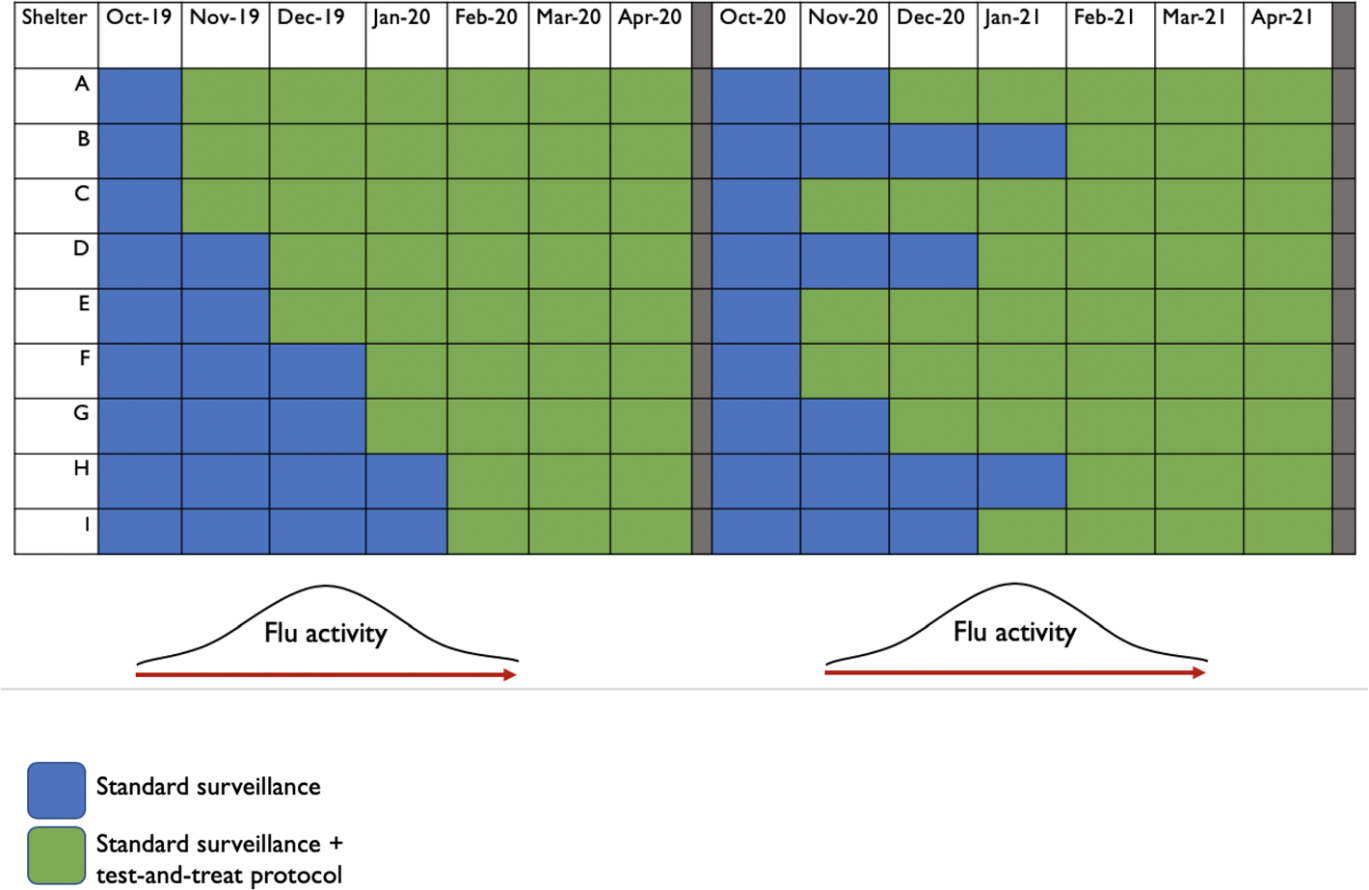
Stepped-wedge design for trial with theoretical influenza (flu) seasons

**Fig. 2:**
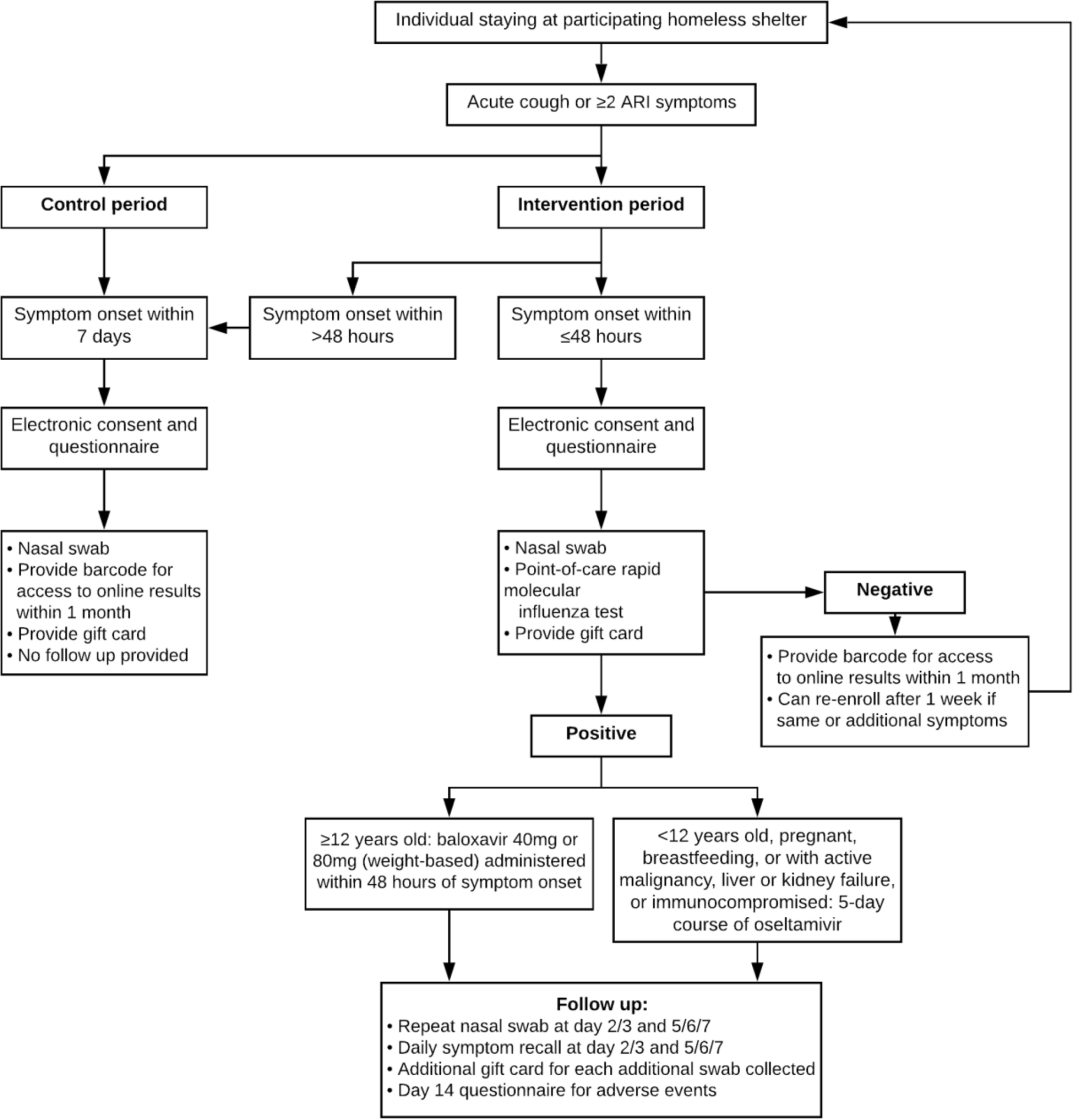
Trial schema

The trial is a stepped-wedge cluster-randomized design clustered by homeless shelter (Fig. 1). The intervention is implementation of on-site point-of-care rapid molecular influenza testing and treatment with baloxavir or oseltamivir for all influenza-positive cases enrolling within 48 h of symptom onset (Fig. 2). Shelters will be randomized to begin the intervention at different months throughout the influenza season. We will conduct this trial over two influenza seasons with re-randomization of the timing of the intervention implementation each season. Individuals within shelters will be eligible to participate if they have two or more qualifying ARI symptoms (see Table 1 for symptom list). The control condition is an influenza-surveillance kiosk installed in a shelter that allows participants to collect a nasal swab that is then sent to a lab for testing. During the intervention period, symptomatic individuals with symptom onset in the prior 48 h who have not yet received antiviral influenza treatment will be eligible for on-site point-of-care rapid molecular influenza testing (Abbott Laboratories, Lake Bluff, IL, USA) at an “improved” kiosk. If individuals do not meet inclusion criteria because of symptom > 48 h duration, they are still eligible for surveillance testing. If intervention-eligible individuals test positive for influenza, they will be administered an antiviral (either baloxavir or oseltamivir based on eligibility criteria). All participants will receive active drug. We will perform whole genome sequencing of influenza-positive samples to evaluate secondary transmission within shelters.

**Table 1:** Acute respiratory illness (ARI) trigger symptoms of which participants should have ≥ 2 to be for eligible for enrollment

Symptoms included in study	
Feeling feverish	Runny or stuffy nose
Headaches	Increased trouble with breathing
Cough^a^	Fatigue (tiredness)
Sore throat or itchy/scratchy throat	Muscle or body aches
Nausea or vomiting	Diarrhea^b^
Rash^b^	Ear pain or ear discharge^b^

^a^New or worsening cough alone fulfills eligibility criteria

^b^Only if under 18 years

Asymptomatic or pauci-symptomatic enrollment: Once a month, there will also be shelter-wide sampling of asymptomatic (i.e., no ARI trigger symptoms, see Table 1) and pauci-symptomatic individuals (i.e., one ARI trigger symptom, excluding new or worsening cough) to estimate the asymptomatic fraction. This will involve enrolling individuals who have been asymptomatic or pauci-symptomatic for the prior 7 days. These individuals will not be eligible for the intervention. They will complete a brief survey and a nasal swab will be collected for on-site influenza testing. Seven days later, they will complete a follow-up survey sent via text message or email to assess for new onset of symptoms in the 7 days following the swab collection. If new symptoms have developed such that participants no longer meet the definition of asymptomatic or pauci-symptomatic, they will not be eligible for inclusion in the asymptomatic fraction.

## Study population

Participants will be any individuals staying at participating shelters. Participants ≥ 12 years of age who test positive for influenza will be given baloxavir as treatment and receive a 7-day follow-up. Participants < 12 years of age and those with active malignancy, liver disease, or immunocompromising condition (see Table 2) who test positive for influenza will receive a 5-day course of oseltamivir.

**Table 2:** Pre-existing conditions that necessitate use of oseltamivir instead of baloxavir

Conditions for which baloxavir contraindicated	
Liver disease	
Cancer	
Immunosuppression (by medication or disease)	
Pregnant or breastfeeding	

### Individual enrollment criteria

Participants must fulfill all the following inclusion criteria:

- Resident for 1 or more days at a participating shelter
- 3 months of age or older
- New or worsening cough, or ≥ 2 ARI symptoms (see Table 1 for list) during past 7 days
- Willingness to take study medication
- Willingness to comply with all study procedures, including weekly surveillance and repeat nasal swab at day 2/3 and day 5/6/7 post-treatment.
- Ability to provide written, informed consent and/or assent

### Exclusion criteria

Individuals meeting any of the following criteria will be excluded:

- Any serious or uncontrolled medical disorder or active infection that, in the opinion of the investigator, may increase the risk associated with study participation or study drug administration
- Inability to consent and/or comply with study protocol
- Receipt of oseltamivir or baloxavir within past 7 days for treatment of influenza
- Known hypersensitivity to baloxavir or oseltamivir
- Chronic kidney disease (CKD) defined as self-reported history of dialysis

### Enrollment criteria for sampling asymptomatic and pauci-symptomatic residents

Participants must fulfill all the following inclusion criteria:

- Resident for 1 or more days at a participating shelter
- 3 months of age or older
- No symptoms or 1 symptom (excluding new or worsening cough) from Table 1 in the prior 7 days
- Willing to comply with all study procedures, including a nasal swab on enrollment, repeat surveillance in 7 days and willingness to repeat nasal swab at day 7 if symptomatic.
- Able to provide written, informed consent and/or assent

### Sample size calculations

The study aims to demonstrate a reduction in influenza incidence after implementation of on-site point-of-care rapid molecular testing for influenza and treatment with baloxavir or oseltamivir. Power calculations were based on an assumed 1.67% incidence rate per month, which was determined based on assumed 12% incidence rate during influenza season.

Assuming nine shelters participating for two seasons each (18 shelter-seasons) and a mean of 200 participants per shelter, we estimate 86% power to detect a risk ratio of 0.50 at a 0.05 two-sided significance level (Additional file 2).

## Study procedures

### Recruitment, screening, and consent

Individuals will be recruited from staffed kiosks at each site and screened for eligibility. Study staff will obtain informed consent from the individual or legal guardian (example form Additional file 3). Once the intervention has been introduced in a shelter, study staff will require the participant to consent to the testing and receipt of the treatment drug in addition to providing questionnaire responses and a nasal swab. To encourage participation, the study team’s presence on site will be advertised, and the study team will be on site at regular days and times. Participants will also be compensated for their time and participation with gift cards.

### Pre-intervention period

During the pre-intervention period, kiosks will screen and enroll individuals for the Seattle Flu Study [26]. Kiosks will be staffed at regular times 6 days a week at each shelter. Individuals with new or worsening cough or ≥ 2 ARI symptoms will be eligible for participation once every 7 days. Eligible individuals who choose to participate will have a mid-turbinate nasal swab (nylon flocked, COPAN FLOQSwab, Murrietta, CA, USA) collected and answer demographic and clinical questions on an electronic tablet using REDCap (Nashville, TN, USA). No on-site testing or treatment will be offered in the shelters during the control period. While not standard in shelters, testing kiosks were selected as the control condition for the pre-intervention period as they are the most efficient and unbiased means of discerning baseline influenza incidence through observational design in an uncontrolled environment within a population that has not sought clinical care for their illness episode.

### Intervention period

Sites will be randomized by an algorithm produced by the study statistician to different starting months for the intervention. This will be concealed from sites until the week of implementation. All sites will remain in the intervention period for the remainder of the season once it has been introduced. Kiosks will continue to be staffed at regular times 6 days a week during the intervention period at each site. Individuals who meet inclusion criteria for the pre-intervention period can continue to enroll. For individuals with symptoms < 48 h, they will be eligible to enroll in the intervention arm. The intervention will include use of an on-site point-of-care rapid molecular influenza test (Abbott ID NOW, Abbott Laboratories, Lake Bluff, IL, USA) which produces a result in 12 min. Baloxavir (XOFLUZA, Genentech, San Francisco, CA, USA) or oseltamivir (TAMIFLU, Roche, Basel, Switzerland) treatment will be administered for all influenza-positive individuals, with pertinent medication counseling provided on-site by kiosk staff. Study clinicians will be available 24/7 by phone to respond to questions or concerns that cannot be directly addressed by the kiosk staff. Influenza rapid test-positive individuals aged 3 months to 11 years, those who are pregnant or breastfeeding, and adults with active malignancy, liver disease, or who are immunocompromised (Table 2) will receive a 5-day supply of oseltamivir (Roche, Basel, Switzerland). All other individuals with influenza-positive results on rapid testing will receive a one-time dose of baloxavir (Genentech, San Francisco, CA, USA). No participants will receive placebo. Individuals who had symptom onset > 48 h before enrollment will be eligible only for routine lab-based testing, as was available during the pre-intervention period.

#### Follow-up

Following antiviral drug receipt, all participants in the intervention arm will be asked to return to the kiosk at their shelter for prospective questionnaire and nasal swab collection on day 2 or 3 and day 5, 6, or 7 after diagnosis (Table 3). Participants will be asked to return a final time on day 14 to report any further adverse events. Kiosk staff will recommend that the parents of all participants aged 3 months to 11 years who test positive for influenza take their child to see their primary care or urgent care provider within 24 to 72 h, depending on age and comorbidities. They will receive a referral letter for their provider and a travel voucher.

**Table 3:** Timing of data collection

	Control	Intervention		
*Data items*	*Enrollment*	*Enrollment (day 0)*	*Follow-up (day 2/3)*	*Follow-up (day 5/6/7)*
Informed consent	X	X		
Participant’s demographic and SES characteristic	X	X		
Clinical data and health-seeking behaviors	X	X	X	X
Nasal swab collection	X	X	X	X
Molecular test		X		
Initial gift card	X	X		
**If positive**				
Antiviral dispensation log	na	X		
Daily symptom questionnaire	na		X	X
Additional $30 gift card	na	X		
Additional $5 gift cards	na	X	X	X

*Abbreviations*: *na* not applicable, *SES* socioeconomic status

Considering the transient nature of this population, we will encourage follow-up through autogenerated text-message reminders for those with cell phones and through paper-based appointment slips provided by kiosk study staff at the time of enrollment.

For those that provide consent for release of information, laboratory results will be released to their on-site providers for treatment follow-up.

#### Asymptomatic and pauci-symptomatic sampling

**Fig. 3:**
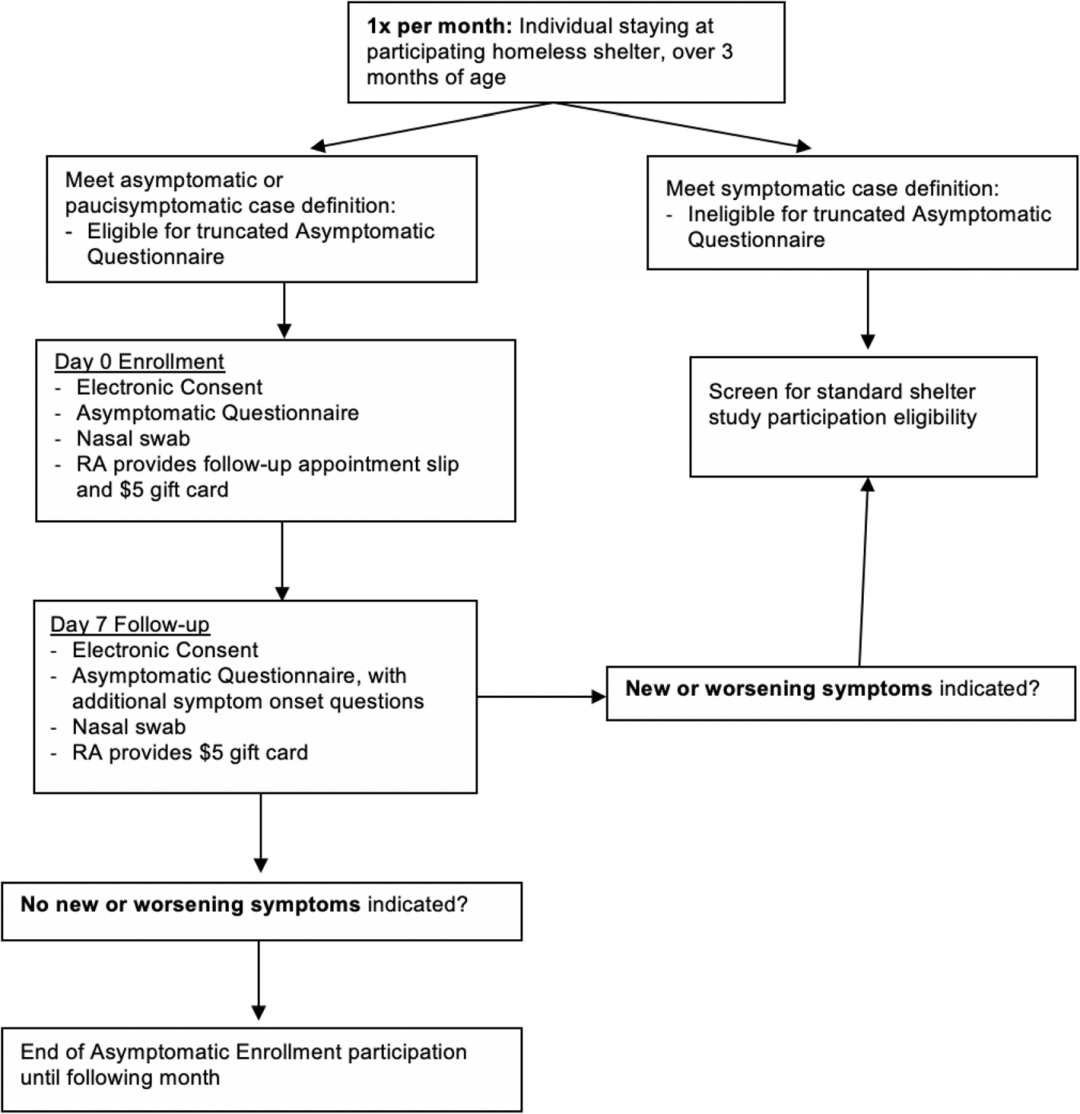
Asymptomatic enrollment schema for study participation November 2019–March 2020. Asymptomatic study participation was modified from a monthly activity to every day there was a research assistant on-site conducting study recruitment and enrollments at a shelter

Once a month, all shelter residents with less than two ARI symptoms (excluding new or worsening cough) will be eligible to participate in the study through collection of a surveillance nasal swab tested via lab-based qRT-PCR and condensed questionnaire (Fig. 3). They will be asked to follow-up on day 7 after the initial swab for a repeat questionnaire. These questionnaires will be sent in autogenerated text messages or by email. If they have developed two or more ARI symptoms in the intervening 7 days, they will be asked for a repeat nasal swab and screened for enrollment into the symptomatic surveillance arm of the study. These specimens will be tested in the same manner as pre-intervention surveillance specimens from symptomatic participants.

## Biospecimen management

Nasal swabs will be placed in universal viral transport media (Beckton Dickinson, Franklin, NJ) and transported at room temperature to University of Washington, where they will be aliquoted in triplicate, barcoded using a unique identifier linked back to the participant and site of collection, and stored at 4 °C until testing. Residual samples will be stored at − 80 °C.

### Laboratory testing

Total nucleic acids will be extracted using the Magna Pure 96 kit (Roche). They will then be tested by TaqMan Open Array RT-PCR (Thermo) for 26 respiratory pathogens. Viral genome sequencing by hybrid capture will be attempted on all influenza-positive samples with viral loads > ~ 50,000 genomic copies/mL using a protocol described previously for the Seattle Flu Study [26].

## Data management

### Data security and privacy

All information from the study subjects will be kept confidential. All forms and specimens will have a participant identification number, given to the participant upon enrollment in the study. Data will be collected electronically in REDCap (Nashville, TN, USA). The REDCap survey app is Title 21 CFR Part 11 compliant, password protected, and an auditable database. The list linking the participant to the ID number will be stored separately from the REDCap database. Access to identifiable information will be limited to the study staff and the study pharmacists (for drug dispensing and delivery purposes). Any datasets that include identifiable information will be stored in a HIPAA-compliant manner via OneDrive for Business (Microsoft, Redmond, WA, USA) at the University of Washington. No identifying information will be included on any data sent to the broader study team or any other data-sharing repositories.

All subjects will also be asked to approve the storage of their biospecimens during the initial consenting process and prior to study participation. Persons who consent to the trial but who do not want their biospecimens stored may still participate. Their biospecimens will be tested per protocol, but remaining aliquots will be destroyed.

### Data quality

Clinical research staff will check data for missing or unusual values and for consistency within participants and shelters in the centralized data capture system. Computerized checks will be conducted daily for any enrollments made to identify missing, inconsistent, or out of range data. Any suspect data will be raised as data queries.

The study coordinator will investigate data queries to provide an explanation and possible resolution of discrepancies on a weekly basis using the data quality module overview on REDCap. The study coordinator will raise queries and share them with the study staff who are involved in the shelter enrollment process or are involved in data collection and management. The study staff will contact participants via their preferred method of communication (phone, in-person, or email) to clarify instances of suspect data. Following this communication, the data items will be marked as “verified,” and an additional review will be conducted by the study coordinator. The query will then be closed. When there are no longer any open queries on a survey, it can be locked by the study coordinator.

A trial steering committee or data quality committee were not deemed necessary for the purposes of this trial since the nature of the RCT is to assess the feasibility, acceptability, and population-level impact of an FDA-approved diagnostic tool and non-experimental, standardized antiviral treatment. Overview and accountability do not require the formulation of additional committees since the nature of the study design results in the majority of participant encounters being observational (only involving a one-time completion of the demographic questionnaire and nasal swab collection) and require no follow-up. Those that do meet the criteria for follow-up protocol adherence have a short follow-up period (7 days) and require their presence at the study site of initial enrollment when they received a positive rapid flu test result. Protocol adherence is thus manageable to evaluate through the tracking of expected follow-up study visits completed in this 1 week period by study coordinators with REDCap and research assistants’ physical presence 6 days per week at study sites.

Similarly, study coordinators are easily able to check on shelters’ recruitment progress through regular REDCap database checks, weekly calls with on-site research assistants conducting enrollments, consistent email correspondence with shelter management, and regular visits to study shelter sites. Staff training is facilitated by coordinators following content review and approval by the study’s primary investigator and co-investigators that hold medical licenses. Post-tests, refresher trainings, and on-site job checks are also conducted by coordinators with all research assistants following trainings to ensure their ability to safely and knowledgeably adhere to study protocols.

### Plan for reporting unanticipated problems/adverse events

On day 2 or 3 and day 5, 6, or 7 following receipt of the study drug, participants will be asked to provide additional nasal swab specimens tested at University of Washington via qRT-PCR and symptom logs. In addition, they will complete questionnaires pertaining to short-term reactions to the study drug that may fit the definition of an adverse event (AE). Any common treatment side effects experienced by the participant, which will have been explained by study staff during the drug counseling process, will be noted in these questionnaires during the follow-up period alongside notes regarding relevant symptoms and their severity. A questionnaire to make a final assessment of AEs and serious adverse events (SAEs) will be administered on day 14. There are no expected adverse events from the antiviral treatments provided in the intervention that are specific to this population. Both oseltamivir and baloxavir are considered standard-of-care medications for influenza and are well tolerated by most patients.

Reported adverse events will be reviewed to determine the relationship to drug treatment and whether it was unexpected. If the event is possibly related to the study, a report will be made to the independent safety officer, who will make a determination. All SAEs will be reported to the data safety and monitoring board (DSMB) regardless of cause. Only events that meet the definition of an SAE will be reported in trial publications.

### Protections against risks

To reduce distress, participants may skip any questions they are uncomfortable answering. Additionally, all participants will be reminded that involvement in research is always optional, and they may terminate their participation at any time without consequences. Neither shelter staff nor the study funder will have access to an individual participant’s data or test results. If the trial intervention proves effective, there is no current plan to continue access to on-site testing and treatment after the trial ends.

### Real-time results

Subjects undergoing influenza point-of-care testing will be immediately notified of their influenza status. All participants, regardless of intervention status at their shelter of residence, will be able to access additional testing results from the study lab on an online platform within 1 month of enrollment. Reported pathogens may include influenza viruses and/or RSV. An access code or URL to view results will be provided by kiosk staff to the participant at the end of their study encounter.

### Future use

Samples will be stored at the University of Washington for future testing.

Deidentified aggregate data will be available to the public on www.seattleflu.org, as will statistical code.

## End of trial

The end of trial is defined as when the last individual has had their last data collected following two subsequent influenza seasons; active participant enrollment will take place over the course of 2 years between October 2019 and is projected to end in May 2021.

## Statistical analyses

Analyses will be intention-to-treat (ITT), consisting of all individuals who have consented to receive an antiviral or who would have been eligible to receive an antiviral had they tested positive or been in the intervention group. Analyses will be based on complete cases. Flu-positive participants who do not complete all follow-up surveys will be treated as uniformly censored as of their most recent valid nasal swab result and will be excluded.

The primary endpoint is as follows: incidence of influenza virus infection will be analyzed using a generalized linear model following a Poisson distribution with a log link and robust variance adjusted for calendar time with an offset of shelter person-days and random effect for shelter. Additional shelter-level covariates (i.e., adult vs. family shelter) may be included in the model to increase precision. This model will be used to estimate/summarize incidence density, a function of number of cases of laboratory-confirmed influenza cases among shelter residents divided by total person-days of all individuals staying at the shelter. Study arms will be compared using a two-tailed Wald test. Time-averaged estimated intervention effect, 95% confidence intervals, and *p* values will be calculated.

For the secondary endpoints of symptom duration and symptom severity, we will use generalized linear mixed effects regression models with logit link functions, adjusted for individual-level variables and time points. Duration will be based on the outcome of influenza viral detection 1 week after diagnosis (yes/no). Severity will be dichotomized into severe or not severe based on the symptom severity 3-point Likert scale in questionnaire results.

For the secondary endpoint of asymptomatic fraction, we will calculate this as the number of asymptomatic and pauci-symptomatic participants with confirmed asymptomatic or pauci-symptomatic status on follow-up divided by the number of eligible participants enrolled on the day of sampling.

### Interim analysis

The primary outcome data will not be analyzed at an interim point, but the secondary outcome data regarding emergence of antiviral resistance in participants who received either baloxavir or oseltamivir will be reviewed at the end of the first year’s influenza season. If observed emerging antiviral resistance exceeds 20%, we will cease participant enrollment in this clinical trial.

### Health resource utilization analysis

The resource utilization analysis will include a “within-trial” cost-effectiveness analysis to compare the costs and the number of missed school or work days accrued over the follow-up period for shelters and individuals in the intervention and control periods attributed to influenza virus infection. Results will be presented as a ratio of the incremental cost per day of school or work missed with point-of-care testing and treatment compared with routine lab-based testing without on-site treatment.

### Translational analysis

Samples will be sent to the central laboratory for storage and analysis. The goals of the translational research will be to determine the association between secondary transmission and (1) influenza virus strain (by genetic sequencing) and (2) viral kinetics (maximum viral load, duration of shedding). The lab will also monitor for the emergence of antiviral resistance among treated cases through identification of PA/I38X and non-PA/138 substitutions for baloxavir and H275Y and other NA mutations for oseltamivir [23, 24].

### Subgroup analyses

Subgroup analyses will be performed to compare influenza incidence rates in youth and family vs. adult shelters. The outcome will be the same as the primary analysis (number of influenza-positive tests).

Results will be disseminated through peer-reviewed publications, meetings, and the SFS website (SeattleFlu.org). Authorship will follow criteria of the International Committee of Medical Journal Editors (ICMJE). Modifications to the protocol will be documented in publications and communicated to trial sites through direct contact with the site supervisor.

## Data safety and monitoring

The study will have a Data and Safety Monitoring Board (DSMB), which will meet prior to the start of subject enrollment and annually to review data from the study. It will be comprised of individuals with expertise in biostatistics, epidemiology, and clinical infectious diseases. The DSBM is responsible for determining if there are problems relating to the safety of the intervention and whether the trial should be stopped. Stopping considerations for the DSMB to recommend terminating the study at the interim analysis (after season 1) include (1) efficacy-based stopping rule based on an O’Brien-Fleming type boundary with alpha = 0.025, (2) operational futility stopping rule if low influenza rate or enrollment rate, or (3) detection of baloxavir resistance and/or transmission of baloxavir-resistant influenza strains. In addition, this study will employ an independent monitor to provide feedback to the investigative team on compliance with protocol and documentation of any protocol violations.

## SARS-CoV-2 pandemic-related modifications

As a result of the SARS-CoV-2 pandemic, year 1 of the trial’s intervention was suspended 1 month early on April 1, 2020, until the following flu season. Due to the potential value of data that may continue to be collected directly from shelters to better understand this novel pathogen, the study was modified to revert back to the pre-intervention study period from April 1, 2020–October 31, 2020, when participant recruitment and data collection was previously unplanned at all nine shelters. Additionally, shelter staff working at these study sites became eligible for standard surveillance study participation (clinical and sociodemographic data collected via tablet-based questionnaire and swab collection). From April 1, 2020, onwards, the study was also modified for asymptomatic and pauci-symptomatic individuals to be eligible for standard surveillance study participation any time there was a study staff on site recruiting and enrolling participants. This change was made to improve early detection of asymptomatic or pre-symptomatic SARS-CoV-2 cases. Eligibility criteria (specifically being a shelter resident and presenting with ARI trigger symptoms) for the trial’s intervention have not changed (see the “Study population” section). Asymptomatic or pauci-symptomatic individuals and shelter staff may participate by providing a completed questionnaire and nasal swab specimen at any time throughout the duration of the study period. However, they are never eligible for the on-site testing and treatment trial.

Following the early suspension of the trial in year 1 and as a reaction to the ongoing pandemic, three participating shelters were closed and moved residents to new physical locations in an effort to reduce crowding in congregate sleeping spaces. Year 2 of the trial will therefore include nine shelters that have maximum nightly populations ranging from 45 to 275 individuals each, with a total maximum nightly population estimated at 1115 individuals. RSV and influenza return of results within 1 month of study participation on an online platform will also no longer be available.

## Supplementary Information

**Additional file 1.** SPIRIT 2013 Checklist: Recommended items to address in a clinical trial protocol and related documents*.

**Additional file 2.** Sample size calculations.

**Additional file 3.** Consent Form.

## Data Availability

Deidentified aggregate data will be available to the public on www.seattleflu.org, as will statistical code.
