# Supplementary material for: Point-of-care molecular testing and antiviral treatment of influenza in residents of homeless shelters in Seattle, WA: study protocol for a stepped-wedge cluster-randomized controlled trial
Source: Trials. 2020 Nov 23;21:956. doi: 10.1186/s13063-020-04871-5 (PMC7682130; doi:10.1186/s13063-020-04871-5)
Supplement: Supplementary file 2 — Additional file 2. Sample size calculations. [file 13063_2020_4871_MOESM2_ESM.docx]

**APPENDIX 2**

Power Calculations

In all calculations, we assume a two-sided test with α = 0.05. Influenza seasons are 6 months. In the general population, influenza incidence is expected to be 10%/season. We believe incidence will be higher in the homeless shelter population and assume incidence of 12%/season or, on average, about 2%/month. Shelters are each expected to contribute 200 person-months of observation and we assume that we will detect 80% of influenza cases at each shelter, so the detected incidence rate will be about 1.6%/month. These assumptions imply, in the absence of an intervention, about 3 detected cases of influenza per shelter per month (200*.02*.8).  Multiple seasons are considered independent replicates, equivalent to recruiting new shelters.

| Power (%) to detect intervention effect as a function of RR and number of shelter-seasons. Assumptions: Six month influenza season, average shelter census = 200, effective control incidence rate = 1.6%/month (=2%*.8), SD(shelter) = .0028, SD(shelter*month) = .0032, SD(intervention) = 0. | | | | | | |
| --- | --- | --- | --- | --- | --- | --- |
|  |  | Number of shelter-seasons | | | | |
|  |  | 8 | 12 | 16 | 18 | 24 |
| RR | 0.7 | 21 | 31 | 36 | 41 | 51 |
|  | 0.65 | 27 | 40 | 48 | 53 | 65 |
|  | 0.6 | 34 | 51 | 60 | 66 | 77 |
|  | 0.5 | 52 | 72 | 81 | 86 | 93 |

Note: Simulations suggest a small loss of power (3-4%) if the incidence rate is not constant over the influenza season
