# Supplementary material for: Point-of-care molecular testing and antiviral treatment of influenza in residents of homeless shelters in Seattle, WA: study protocol for a stepped-wedge cluster-randomized controlled trial
Source: Trials. 2020 Nov 23;21:956. doi: 10.1186/s13063-020-04871-5 (PMC7682130; doi:10.1186/s13063-020-04871-5)
Supplement: Supplementary file 3 — Additional file 3. Consent Form. [file 13063_2020_4871_MOESM3_ESM.pdf]

Consent Form

UNIVERSITY OF WASHINGTON CONSENT FORM: Ages 18 and up ASSENT FORM: Ages 13-17 SEATTLE FLU STUDY - The Shelter Study

Researchers: Helen Y. Chu, MD, MPH, Assistant Professor of Medicine (University of Washington)
Janet A. Englund, MD, Professor of Pediatric Infectious Diseases (Seattle Children's Hospital)
Michael Boeckh, MD, PhD, Professor of Medicine (Fred Hutchinson Cancer Research Center)
To contact a research staff during regular business hours, M-F 9:00AM - 5:00PM: 206-685-5386
To contact a study clinical provider during an emergency, available 24-hours a day: (206) 598-4846

We are asking you to be in a research study. This form gives you information to help you decide whether or not to be in the study. Being in the study is voluntary. Please read this carefully. You may ask any questions about the study. Then you can decide whether or not you want to be in the study.

This form also serves as an assent form for individuals age 13-17. This means that if you choose to take part in this research study, you would sign this form to confirm your choice. Your parent or legally-authorized representative would also need to give their permission and sign this form for you to join the study.

This form is also used for parents and legally-authorized representatives of subjects to provide permission to the research team for individuals who are not capable of providing permission themselves. In such cases, the term "you" refers to the subject or your child.

Purpose of the Study The flu and colds are common in children and adults, and can cause a runny nose, cough or fever. We want to understand how people get sick with these types of sickness and how they spread from person to person. Once we understand how the germs start and spread, we can have a better plan to prevent them from making people sick. This study will look at whether testing and treating people that are sick with the flu in shelters help people recover faster from their illness and if it prevents other people staying at the shelter from getting sick as well. We also want to provide people with a way to see what germs are in their neighborhood. This information may be on the internet for everyone to see.

Seattle Flu Study researchers, Washington State Department of Health, and the Centers for Disease Control and Prevention (CDC) are closely monitoring an outbreak of respiratory disease. This outbreak is caused by a novel (new) coronavirus. Some person-to-person spread of this virus outside China has been detected. Because of your symptoms today, we would like to test to see if you (or your child) have an infection caused by this new coronavirus. This information will not be on the internet for everyone to see.

Study Procedures We are testing children and adults who might have the flu or cold. We are inviting you to participate because you have the symptoms of the flu or cold. This study involves:
Answering questions about your health, who you are around, and your living situation. There will also be questions about smoking, alcohol and IV drug use. We think these questions will take about 15 minutes. You may choose not to answer any question that you do not want to. Doing a swab of your nose to look for germs. This will only take about 5 seconds. To test for novel coronavirus, the swab may be sent to a public health laboratory for testing with a test that was authorized for emergency use. If the test is positive, you may be contacted by your local health department, You might be asked to restrict your (your child's) movement. This may mean that you (your child) would need to stay away from other people until the infection has cleared. Also you might be asked about who you (your child) has been in contact with since becoming ill. The people you (your child) has been in contact with might be contacted to see if they are ill too. You may also be asked to provide additional swabs for testing. You may not be contacted if the first test result is negative. We will ask your permission to see your medical records if you get very sick and have to go to the hospital. This will help us learn whether you got sick from the flu or from something else. Testing your nose swab right away using a machine. This should take around 30 minutes. If you have had symptoms for 48 hours or less, and you test positive for the flu, we will be giving you a one-time dose of an antiviral medicine. If your flu test is positive and you have had symptoms for 48 hours or less:
Children under 12 years of age, or weighing less than 88 pounds, will be given a medicine called oseltamivir. Adults who are pregnant or who have serious medical conditions will also receive oseltamivir. Children 12 or older, and adults without serious medical comorbidities, will receive a medicine called baloxavir marboxil. Both of these antivirals have been approved by the Food and Drug Administration and are standard treatment for flu. We will then ask you to come back to our study team in the same spot two more times in the next week for your nose to be swabbed again and to answer just a few questions about how you are feeling. This study does not replace care you would receive from your doctor, and your participation is voluntary. The only test for this study is the swab of your nose.

Risks, Stress, or Discomfort We do not expect serious side effects from this study. Some rare side effects of baloxavir marboxil include: diarrhea; bronchitis; nausea; nasopharyngitis; and headaches. Side effects of oseltamivir include nausea, vomiting, stomach ache, nosebleeds, headache, and feeling tired. Very rarely, people can have a severe blistering skin rash after taking this medicine.
If you become injured or ill as a result of this study, and require medical care, the cost of the treatment may be billed to you or your health insurance just like other medical costs, or it may be covered by the UW's discretionary Human Subjects Assistance Program (HSAP), depending on a number of factors. The researcher may request HSAP coverage by following established medical procedures. If you wish to request HSAP coverage yourself, contact the researcher or the UW Human Subjects Division at 206-221-5940 if you do no totherwise have access to a telephone. Ask the researcher if you would like information about the limits and conditions of the HSAP. The UW does not normally provide any form of compensation for injury. However, the law may allow you to seek payment for injury-related expenses if they are caused by malpractice or the fault of the researcher. You do not waive any right to seek payment by signing this consent form.
Getting the nasal swab may cause mild discomfort, watery eyes, or sneezing. Some of the questions that we might ask you are sensitive and may make you feel uncomfortable. You do not have to answer any questions that you do not want to. There is a risk that your privacy could be breached. We will do everything that we can to make sure that this does not happen.

Alternative to Taking Part in This Study If you are having symptoms of cold or flu but do not wish to participate in this study, a member of the study staff will share with you a list of alternative means of receiving care and treatment, whether that is a list of clinics nearby or if there are operating clinic hours at your shelter of residence. Study staff will provide witht he steps required to access this care.

Benefits of the Study You will only benefit from free testing and treatment of the flu by participating in this study. It may help others in the future by learning what germs make people sick and how to prevent these germs from spreading to others. Results from the novel coronavirus test could help identify spread of the virus in the community.

Source of Funding The University of Washington is receiving financial support from an anonymous donor.

Confidentiality of Research Information Your record will be kept secret. You will not be identified in any report about this study. All records will be kept in a secure location at the University of Washington. Your study record will never be used against you.
The information we collect from you and the germs that make you sick will be on a public website to show the spread of germs in your community. This infromation will not be shared in a way that will reveal your identity,
The persons involved in these novel coronavirus testing activities and the state or local health department will know about your (your child's) personal information and the results of testing. Beyond this, your name (and your child's name) will be kept confidential and will not be shared.
Researchers at the University of Washington and Fred Hutchinson Cancer Research Center will have access to your data from the study in order to analyze the results.
Government and university staff sometimes review studies such as this one to make sure they are being done safely and legally. If a review of this study takes place, your records may be examined. The reviewers will protect your privacy. The study records will never be used against you.
The U.S. Food and Drug Administration (FDA) reserves the right to review study data that may contain identifying

information.
A description of this clinical trial will be available on http://www.ClinicalTrials.gov, as required by U.S. Law. This Web site will not include information that can identify you. At most, the Web site will include a summary of the results. You can search this Web site at any time.
Washington State law requires that we report certain conditions to local public health jurisdictions or the Department of Health. If your sample tests positive for one of these conditions, a local health jurisdiction or the Department of Health might contact you with more questions. They will keep your information secret.

Use of Information and Specimens Characterizing Germs
We will characterize the germs that are making you sick and share information about them. Information that identifies you would not be shared on the internet.
Returning Results to You
You will receive a link to a website where you can view the results of your nose swab test within three months of when the swab is collected. You can enter your nose swab barcode to view your results for influenza (flu) testing when they are ready. The results may tell you what germs are making you sick, how they are characterized, and advice on how to keep other people from getting sick. You are not required to go to the website and look at your results if you do not want to. We may not be able to give you your results if you lose your barcode.

Using Your Data in Future Research
Storing samples so researchers can use them in the future is calling “banking.” Researchers also bank information and samples so they can share it with other researchers.
The information and/or specimens that we obtain from you for this study might be used for future studies. We may remove anything that might identify you from the information and specimens. If we do so, that information and specimens may then be used for future research studies or given to another investigator without getting additional permission from you. It is also possible that in the future we may want to use or share study information that might identify you. If we do, a review board will decide whether or not we need to get additional permission from you. It is possible the information will be used in future public health investigations. However, these will NOT include human genetic analysis.
By signing this consent form, you are giving your permission for these researchers to use your sample for future use.

Commercial Profit

Your data and/or samples may be used to make new products, tests, or findings. These may have value and may be developed and owned by the research team and/or others. If this happens, there are no plans to pay you.

Other Information You may refuse to participate and you are free to withdraw from this study at any time without penalty or loss of benefits to which you are otherwise entitled. Please note you have the option to accept or refuse this novel coronavirus testing.
If you join this study, you will receive a $5 gift card for each nose swab that we collect. You will receive the gift card the same day that we collect the sample.
If you test positive for flu and take the study flu medicine, you will receive a $30 gift card.

Research-Related Injury If you think you have been harmed from being in this research, or have a medical problem or illness related to this research, contact our research staff at (206) 598-4846 right away. He or she will treat you or refer you for treatment. The University of Washington does not normally provide compensation for harm except through its discretionary program for medical industry. However, the law may allow you to seek payment by signing this consent form.

If you become injured or ill as a result of this study, and require medical care, the costs of the treatment may be billed to you or your health insurance just like other medical costs, or it may be covered by the UW’s discretionary Human Subjects Assistance Program (HSAP), depending on a number of factors. The researcher may request HSAP coverage by following established procedures. If you wish to request HSAP coverage yourself, contact the researcher or the UW Human Subjects Division at hsdinfo@uw.edu or 206-543-0098. You may also call collect to the UW Human Subjects Division at 206-221-5940 if you do not otherwise have access to a telephone. Ask the researcher if you would like information about the limits and conditions of the HSAP. The UW does not normally provide any other form of compensation for injury. However, the law may allow you to seek payment for injury-related expenses if they are caused by malpractice or the fault of the researchers. You do not waive any right to seek payment by signing this consent form.

Study Procedures We are testing children and adults who might have the flu or cold. We are inviting you to participate because you have the symptoms of the flu or cold. This study involves:
Answering questions about your health, who you are around, and your living situation. There will also be questions about smoking, alcohol and IV drug use. We think these questions will take about 15 minutes. You may choose not to answer any question that you do not want to. Doing a swab of your nose to look for germs. This will only take about 5 seconds. To test for novel coronavirus, the swab may be sent to a public health laboratory for testing with a test that was authorized for emergency use. If the test is positive, you may be contacted by your local health department. You might be asked to restrict your (your child's) movement. This may mean that you (your child) would need to stay away from other people until the infection has cleared. Also, you might be asked about who you (your child) has been in contact with might be contacted to see if they are ill too. You may also be asked to provide additional swabs for testing. You may not be contacted if the frist result is negative. We will ask your permission to see your medical records if you get very sick and have to go to the hospital. This will help us learn whether you got sick from the flu or from something else. This study does not replace care you would receive from your doctor, and your participation is voluntary. The only test for this study is the swab of your nose.

Risks, Stress, or Discomfort We do not expect serious side effects from this study. Getting the nasal swab may cause mild discomfort, watery eyes, or sneezing. Some of the questions that we might ask you are sensitive and may make you feel uncomfortable. You do not have to answer any question that you do not want to. There is a risk that your privacy could be breached. We will do everything that we can to make sure that this does not happen.

Benefits of the Study You will not benefit directly from this study. It may help others in the future by learning what germs make people sick and how to prevent these germs from spreading to others. Results from the novel coronavirus testing could help identify spread of the virus in the community.

Source of Funding The University of Washington is receiving financial support from an anonymous donor.

Confidentiality of Research Information Your record will be kept secret. You will not be identified in any report about this study. All records will be kept in a secure location at the University of Washington. Your study record will never be used against you.
Researchers at the University of Washington and Fred Hutchinson Cancer Research Center will have access to your data from the study in order to analyze the results.
The information we collect from you and the germs that make you sick will be on a public website to show the spread of germs in your community. This information will not be shared in a way that will reveal your identity.
The persons involved in these novel coronavirus testing activities and the state or local health department will know about your personal (your child's information and the results of testing. Beyond this, your name (and your child's name) will be kept confidential and will not be shared.
Government and university staff sometimes review studies such as this one to make sure they are being done safely and legally. If a review of this study takes place, your records may be examined. The reviewers will protect your privacy. The study records will never be used against you.
The U.S. Food and Drug Administration (FDA) reserves the right to review study data that may contain identifying information.
A description of this clinical trial will be available on http://www.ClinicalTrials.gov, as required by U.S. Law. This Web site will not include information that can identify you. At most, the Web site will include a summary of the results. You can search this Web site at any time.
Washington State law requires that we report certain conditions to local public health jurisdictions or the Department of Health. If your sample tests positive for one of these conditions, a local health jurisdiction or the Department of Health might contact you with more questions. They will keep your information secret.

Use of Information and Specimens Characterizing Germs
We will characterize the germs that are making you sick and share information about them. Information that identifies you would not be shared on the internet.
Using Your Data in Future Research
Storing samples so researchers can use them in the future is calling “banking.” Researchers also bank information and samples so they can share it with other researchers.
The information and/or specimens that we obtain from you for this study might be used for future studies. We may remove anything that might identify you from the information and specimens. If we do so, that information and specimens may then be used for future research studies or given to another investigator without getting additional permission from you. It is also possible that in the future we may want to use or share study information that might identify you. If we do, a review board will decide whether or not we need to get additional permission from you. It is possible the information will be used in future public health investigations. However, these will NOT include human genetic analysis.
By signing this consent form, you are giving your permission for these researchers to use your sample for future use.

Commercial Profit
Your data and/or samples may be used to make new products, tests, or findings. These may have value and may be developed and owned by the research team and/or others. If this happens, there are no plans to pay you.

Other Information You may refuse to participate and you are free to withdraw from this study at any time without

penalty or loss of benefits to which you are otherwise entitled. Please note you have the option to accept or refuse this novel coronavirus testing.
If you join this study, you will receive a $5 gift card for each nose swab that we collect. You will receive the gift card the same day that we collect the sample.

Research-Related Injury If you think you have been harmed from being in this research, or have a medical problem or illness related to this research, contact our research staff at (206) 598-4846 right away. He or she will treat you or refer you for treatment. The University of Washington does not normally provide compensation for harm except through its discretionary program for medical industry. However, the law may allow you to seek payment by signing this consent form.

Subject’s statement
This study has been explained to me. I volunteer to take part in this research. I have had a chance to ask questions. If I have questions later about the research, or if I have been harmed by participating in this study, I can contact one of the researchers listed on the first page of this consent form. I give permission to the researchers to use my medical records as described in this consent form. If I have questions about my rights as a research subject, I can call the Human Subjects Division at (206) 543-0098 or call collect at (206) 221-5940. I will receive a copy of this consent form.

Subject's Statement This study has been explained to me. I volunteer to take part in this research. I have had a chance to ask questions. If I have questions later about the research, or if I have been harmed by participating in this study, I can contact one of the researchers listed on the first page of this consent form. If I have any questions about my rights as a research subject, I can call the Human Subjects Division at (206) 543-0098 or call collect at (206) 221-5940. I will receive a copy of this consent form.

Participant's First Name: ______________________________

Participant's Last Name: ______________________________

First name of [participant_first_name]'s parent/legally authorized representative: ______________________________

Last name of [participant_first_name]'s parent/legally authorized representative: ______________________________

Signature of [participant_first_name]'s parent/legally authorized representative:

____

UNIVERSITY OF WASHINGTON ASSENT TO RESEARCH: Ages 7-12 SEATTLE FLU STUDY - The Shelter Study
Researchers: Helen Y. Chu, MD, MPH, Assistant Professor of Medicine (University of Washington)
Janet A. Englund, MD, Professor of Pediatric Infectious Diseases (Seattle Children's Hospital)
Michael Boeckh, MD, PhD, Professor of Medicine (Fred Hutchinson Cancer Research Center)
To contact research staff during regular business hours, M-F 9:00AM – 5:00PM: (206) 685-5386
To contact a study clinical provider during an emergency, available 24-hours a day: (206) 598-4846

Researcher's statement:
We are asking you to be in a research study because you have the symptoms of a cold such as a fever, runny nose and cough. We are trying to learn more about the germs that cause these symptoms. This form gives you information to help you decide whether or not to be in the study. Please read this carefully. You may ask any questions about the study. Then you can decide whether or not you want to be in the study.
Seattle Flu Study researchers, Washington State Department of Health, and the Centers for Disease Control and Prevention (CDC) are closely monitoring an outbreak of respiratory disease. This outbreak is caused by a novel (new) coronavirus. Some person-to-person spread of this virus outside China has been detected. Because of your symptoms today, we would like to test to see if you (or your child) have an infection caused by this new coronavirus.
If you choose to be in the research, we would ask you to do the following:
Questions: A person on the research team would ask you and/or your parents some questions. You or your parents do not have to answer any question you don't want to. Nasal swab: We would take a little bit of snot from your nose by putting a swab (like a Q-tip) in your nose and twirling it around. To test for novel coronavirus, the swab may be sent to a public health laboratory. If the test is positive, you may be contacted by your local health department. You might be asked to restrict your movement. This may mean that you (your child) would need to stay away from other people until the infection has cleared. Also, you might be asked about who you have been in contact with since becoming ill. The peopl you (your child) has been in contact with might be contacted to see if they are ill too. You may also be asked to provided additional swabs for testing. You may not be contacted if the first test result is negative. The nose swab test might hurt just a little bit. It might make you feel like you have to sneeze or cough. Some of the questions might make you uncomfortable. You do not have to answer any question you don't want to. This research will not directly help you, although we do hope to learn something from this research. Results from the novel coronavirus test could help identify spread of the virus in the community.
Please talk this over with your parents before you decide whether or not to do this. We will also ask your parents if it is okay for you to be in this study. But even if your parents say "yes" you can still decide not to do this.
If you don't want to be in the study, you don't have to participate. Remember, being in this study is up to you and no one will be upset if you don't want to participate or even if you change your mind later and want to stop.
You can ask any questions about the study. If you have a question later you can call a researcher at (206) 685-8702 or ask us at this spot next time you see us.
Signing your name at the bottom means that you agree to be in this study. You and your parents will be given a copy of this form after you have signed it.

Your statement:
This research has been explained to me. I agree to take part in this study. I have had a chance to ask questions. If I have more questions, I can ask the doctor or researcher.

UNIVERSITY OF WASHINGTON ASSENT TO RESEARCH: Ages 7-12 SEATTLE FLU STUDY - The Shelter Study
Researchers: Helen Y. Chu, MD, MPH, Assistant Professor of Medicine (University of Washington)
Janet A. Englund, MD, Professor of Pediatric Infectious Diseases (Seattle Children's Hospital)
Michael Boeckh, MD, PhD, Professor of Medicine (Fred Hutchinson Cancer Research Center)
To contact research staff during regular business hours, M-F 9:00AM – 5:00PM: (206) 685-5386
To contact a study clinical provider during an emergency, available 24-hours a day: (206) 598-4846

Researcher's statement:
We are asking you to be in a research study because you have the symptoms of a cold such as a fever, runny nose and cough. We are trying to learn more about the germs that cause these symptoms. This form gives you information to help you decide whether or not to be in the study. Please read this carefully. You may ask any questions about the study. Then you can decide whether or not you want to be in the study.
Seattle Flu Study researchers, Washington State Department of Health, and the Centers for Disease Control and Prevention (CDC) are closely monitoring an outbreak of respiratory disease. This outbreak is caused by a novel (new) coronavirus. Some person-to-person spread of this virus outside China has been detected. Because of your symptoms today, we would like to test to see if you (or your child) have an infection caused by this new coronavirus.
If you choose to be in the research, we would ask you to do the following:
Questions: A person on the research team would ask you and/or your parents some questions. You or your parents do not have to answer any question you don't want to. Nasal swab: A little bit of snot would be collected from your nose by putting a swab (like a Q-tip) in your nose and twirling it around. Flu test: We would take some of the snot from your nose to test it for influenza (one of the germs that could cause your symptoms) using our machine. In about 30 minutes, we would be able to tell you and your parents if you tested positive or negative for this germ. To test for novel coronavirus, the swab may be sent to a public health laboratory. If the test is positive, you may be contacted by your local health department. You might be asked to restrict your movement. This may mean that you (your child) would need to stay away from other people until the infection has cleared. Also, you might be asked about who you have been in contact with since becoming ill. The peopl you (your child) has been in contact with might be contacted to see if they are ill too. You may also be asked to provided additional swabs for testing. You may not be contacted if the first test result is negative. If you test positive for flu, and you have been feeling sick for 48 hours or less:
We would give you some medicine to take for 5 days to help get rid of these germs. Two more visits: We would ask you to come back to our study team in the same spot two more times in the next week for your nose to be swabbed again and to answer just a few questions about how you are feeling. The nose swab test might hurt just a little bit. It might make you feel like you have to sneeze or cough. Some of the questions might make you uncomfortable. You do not have to answer any question you don't want to. If you are less than 12 years old and under 88 pounds, we would give a drug called oseltamivir. Once in a while, people who take oseltamivir can feel dizzy, or might feel like they need to throw up. They can also get a stomach ache, nosebleed, headache, or feel tired. Very rarely, people can have a severe rash with blisters after taking this medicine. Kids who are 12 years old or older and weigh 88 pounds or more will get a medicine called baloxavir. Once in a while, people who take baloxavir can get diarrhea or feel like they need to throw up, or can get a headache or runny nose.
This research will give you free medicine that might make you feel better, and otherwise it will not help you. We do hope to learn something from this research. Results from the novel coronavirus test could help identify the spread of the virus in the community.
Please talk this over with your parents before you decide whether or not to do this. We will also ask your parents if it is okay for you to be in this study. But even if your parents say "yes" you can still decide not to do this.
If you don't want to be in the study, you don't have to participate. Remember, being in this study is up to you and no one will be upset if you don't want to participate or even if you change your mind later and want to stop.
You can ask any questions about the study. If you have a question later you can call a researcher at (206) 685-8702 or ask us at this spot next time you see us. If it is an emergency, you can call anytime at (206) 598-4846.

Signing your name at the bottom means that you agree to be in this study. You and your parents will be given a copy of this form after you have signed it.
Your statement:
This research has been explained to me. I agree to take part in this study. I have had a chance to ask questions. If I have more questions, I can ask the doctor or researcher.

Consent Date ______________________________

Participant's Signature: ______________________________

We would like to email you. If you choose to enroll, we will use this email address to:
- Send you a copy of your consent form
- Ask you follow-up questions about your illness
- Send you your sample barcode so you can view your test results online
- Send you more information about this study and related topics

You may opt out of receiving emails at any time. We will not share your email with anyone outside of the study team.

____
(Ex: myemail@email.com)
